# Observation of inflammation and macrophage polarization in an experimental model of cigarette smoke-exposed mice and cigarette smoke extract (CSE)-induced RAW264.7 cells: An experimental preclinical study

**DOI:** 10.18332/tid/221091

**Published:** 2026-07-26

**Authors:** Jiazhen Zhao, Yang Liu, Yaxi Li, Zhiyuan Liu, Min Tan, Changhui Wang, Xuan Long, Xiaolian Song

**Affiliations:** 1 The First Affiliated Hospital of Anhui University of Science & Technology, Huainan, China; 2 Department of Respiratory and Critical Care Medicine, Shanghai Tenth People’s Hospital, Tongji University School of Medicine, Shanghai, China

**Keywords:** COPD, inflammation, macrophage polarization, cigarette smoke, cigarette smoke extract

## Abstract

**INTRODUCTION:**

The present study aimed to investigate macrophage polarization imbalance and its association with sustained NF-κB/NLRP3 signaling under chronic inflammatory stimulation using both *in vivo* and in vitro COPD-related models.

**METHODS:**

This experimental preclinical study was conducted between March and September 2023, at Shanghai Tenth People's Hospital. A chronic obstructive pulmonary disease (COPD)-like inflammatory model was established in C57BL/6J mice (n=5 per group) through combined chronic cigarette smoke (CS) exposure (4 hours/day, 5 days/week for 15 weeks) and intranasal lipopolysaccharide (LPS) administration (7.5 μg on days 1 and 14), together with an *in vitro* cigarette smoke extract (CSE)-induced RAW264.7 macrophage model (10% CSE for 24 hours). Macrophage M1/M2 polarization and inflammatory responses were assessed using ELISA, qRT-PCR, immunohistochemistry, Western blotting, and flow cytometry.

**RESULTS:**

Airflow limitation was observed in CS-exposed model mice, with significant reductions in FEV_0.05_/FVC (21.70 ± 0.96 vs 27.50 ± 0.94%, p<0.0001) and FEV_0.1_/FVC (77.39 ± 2.62 vs 87.21 ± 1.95%, p<0.001), along with increased lung resistance (RL: 1.22 ± 0.13 vs 0.97 ± 0.02 cmH_2_O/(mL/s), p<0.01). Histological examination revealed thickened bronchial walls, disrupted alveolar structure, and inflammatory cell infiltration. TNF-α (BALF: 290.6 ± 7.58 vs 125.6 ± 5.30 pg/mL, p<0.0001), IL-6 (BALF: 67.92 ± 3.02 vs 44.42 ± 2.62 pg/mL, p<0.001), and IL-1β (BALF: 120.3 ± 8.24 vs 87.52 ± 3.40 pg/mL, p<0.01) levels increased dramatically in both CS-exposed mice and CSE-induced RAW264.7 cells, whereas IL-10 levels decreased (BALF: 46.12 ± 1.71 vs 77.19 ± 3.96 pg/mL, p<0.001).

**CONCLUSIONS:**

CS and CSE exposure induced M1 macrophage polarization in mice, as well as 10% CSE-induced RAW 264.7 cells, which may be related to the activation of the NF-κB/NLRP3 signaling pathway.

## INTRODUCTION

Chronic obstructive pulmonary disease (COPD) is a progressive respiratory disease characterized by airflow obstruction, airway inflammation, and irreversible changes in the lungs^1-3^. It affects approximately 384 million people worldwide and is projected to become the third leading cause of death by 2030^4^. COPD is a significant public health problem worldwide, with many people having the disease (high prevalence), causing a great deal of suffering due to illness (high morbidity), and contributing to increasing rates of death from COPD worldwide^5,6^. More recent estimates from the Global Burden of Disease Study 2015 indicate that COPD remains a leading cause of mortality, with 3 million deaths annually^1^. The estimated prevalence of COPD will continue to increase in the future. Although cigarette smoking is the primary cause of COPD in most areas of the world, other exposures, such as using biomass fuel, indoor and outdoor air pollution, and occupational irritant exposures, are also contributing factors^3^.

COPD symptoms include gradually worsening difficulty breathing (dyspnea), poor lung function, and reduced quality of life. As the airway becomes damaged, COPD is characterized by ongoing inflammation in the airways and lungs, resulting in narrowed airways, changes to the lung’s alveoli, and ongoing loss of compliance over time^4,5^. However, the exact cellular interactions that determine COPD progression remain poorly understood^8^. Among the immune cells involved in the development of COPD, macrophages are important effectors of pulmonary inflammation^5,7^.

Macrophages regulate immune surveillance, clear debris, and produce inflammatory mediators, and are significantly increased in the lungs of patients with various forms of COPD^5-7^, as such cells produce many types of cytokines, chemokines, and proteolytic enzymes that will stimulate both innate and adaptive immune responses, thereby contributing to ongoing lung inflammation and tissue injury^8^.

The remarkable plasticity of macrophages can also be classified by how they respond to signals from their microenvironments during macrophage polarization. While classically activated macrophages (M1-like) produce proinflammatory cytokines such as interleukin (IL)-1β, tumor necrosis factor-α (TNF-α), and IL-6, alternatively activated macrophages (M2-like) produce anti-inflammatory cytokines such as IL-10, IL-4, transforming growth factor-β (TGF-β), and play a role in tissue repair^6,9-12^.

Furthermore, under persistent environmental (e.g. cigarette smoke) and inflammatory (e.g. active inflammation) stimuli, macrophages within the context of chronic inflammatory diseases such as COPD may actively shift between activation-associated states^12,13^.

The question of how pro-inflammatory and alternatively activated macrophage-associated profiles contribute to the development of COPD remains open to debate: some evidence suggests that cigarette smoke exposure leads to enhanced activation of pro-inflammatory macrophages^12^; other studies show an increased prevalence of alternatively activated macrophage-associated phenotypes in COPD-affected lungs^11^. This evidence makes it clear that there is considerable complexity in macrophage response to environmental factors in patients with COPD; therefore, integrating results from *in vitro* and *in vivo* studies will provide valuable insight into the mechanisms of macrophage activation under prolonged inflammatory conditions.

Another important factor in the progression of COPD is recurrent bacterial infections. These infections significantly contribute to airway inflammation and alter macrophage activation status during acute exacerbations. However, the effect of cigarette smoke exposure on the inflammatory response to bacterial infection and on macrophage phenotype and signaling remains ambiguous.

Therefore, the present study aimed to: 1) characterize macrophage polarization imbalance in a combined CS+LPS-induced COPD mouse model and CSE-stimulated RAW264.7 cells; 2) investigate the association between macrophage polarization and sustained NF-κB/NLRP3 signaling pathway activation; and 3) determine the relationship between *in vitro* and *in vivo* findings to better understand the mechanisms of CS-induced macrophage dysfunction in COPD. Information derived from measurements of pulmonary function, histology, inflammatory cytokine analysis, macrophage surface markers, and NF-κB/NLRP3 signaling will assist in understanding how continued inflammation is associated with imbalanced macrophage activation in COPD-like conditions.

## METHODS

### Experimental design, setting and animal allocation

This experimental preclinical study was conducted between March 2023 and September 2023 at the Department of Respiratory and Critical Care Medicine, Shanghai Tenth People’s Hospital, Tongji University School of Medicine, Shanghai, China. A total of 10 adult-specific pathogen-free, C57BL/6J male mice (25–30 g, 6–8 weeks) were obtained via Shanghai SLAC Laboratory Animal Co., Ltd., and housed in rooms with consistent temperature (22 ± 2°C), humidity (50 ± 10%), and a 12-hour light-dark cycle. Before tests, animals were kept pathogen-free with free food and water for a week. Mice were randomly assigned to two groups: a control (air-exposed) group (n=5) and a cigarette smoke (CS) + LPS-exposed group (n=5). All animals in each group underwent the same exposure protocol for three months. Following the final exposure, mice were used for pulmonary function testing, bronchoalveolar lavage fluid (BALF) collection, serum cytokine analysis, histological assessment, flow cytometry, Western blotting, and qRT-PCR analyses as described below. Due to tissue requirements, not all assays were performed on the same lung lobes; however, identical tissue allocation strategies were applied across all experimental groups.

The ethics committee approved all animal operations (Approval No. SHSY-IEC-4.1/21-51/01) and followed the National Institutes of Health’s Handbook for the Care and Use of Laboratory Animals (NIH) (8th edition, National Academies Press, 2011)6. The ARRIVE checklist is provided in the Supplementary file.

### Cigarette smoke (CS) generation and exposure characterization

We used a combined cigarette smoke (CS) and lipopolysaccharide (LPS) exposure methodology to create a model of chronic obstructive pulmonary disease (COPD) with inflammation. This technique is based on previously published methods, modified with only minor changes^13,14^.

An automated device was used to create CS via an automated exposure method, providing the reproducibility needed to yield an accurate model for studying human respiratory illness. All CS were made from filtered CS produced by commercial cigarettes (11 mg tar, 12 mg CO, 1.0 mg nicotine; Double Happiness Cigarettes, Shanghai Tobacco Group, China). All cigarettes were smoked according to standard methods with fixed puff size, puff duration, and each cigarette was smoked to completion within a designated period of time.

Each exposure session was for 1 hour, with a total of 6 cigarettes being burned during each exposure session. Mice were exposed to CS for four sessions (4 h) per day for each of 5 days per week (15 exposure sessions) for 3 months (15 weeks of exposure). This exposure protocol was selected based on previously validated COPD mouse models demonstrating that this duration and intensity (4 hours/day, 5 days/week for 12–24 weeks) reliably induces stable airflow limitation, chronic inflammatory remodeling, and emphysematous changes characteristic of human COPD^13,14^. The 15-week time point was chosen as it represents an established intermediate-to-late stage of COPD-like pathology, allowing investigation of both established inflammation and macrophage polarization changes. There were resting periods between exposure sessions to allow for animal recovery and chamber ventilation. Each cigarette had diluted air added between cigarettes to prevent smoke build-up. Additionally, every time a cigarette was smoked, a short period of filtered air was allowed into the chamber to minimize excess accumulation of residual cigarette smoke.

The amount of total particulate matter (TPM) in the exposure chamber was measured by gravimetric methods using Pallflex filters. The concentration of carbon monoxide (CO) was continuously measured during the entire exposure through the use of a CO monitor calibrated prior to use. Throughout all studies, average TPM and CO concentrations were kept within the same range among the three exposure sessions as a means of ensuring reproducibility.

Unrestrained mice were exposed in the exposure chamber at the same density for unimpeded and evenly distributed movement to minimize shielding effects (the number of animals per chamber remained the same among all exposure sessions).

In order to induce inflammatory exacerbation mimicking acute bacterial exacerbations in COPD patients, LPS derived from Escherichia coli (*E. coli*) O111:B4 (Sigma-Aldrich Inc, USA) was administered intranasally to experimental mice. This dosing regimen was based on validated COPD exacerbation models^15^, where 7.5 μg LPS represents a moderate inflammatory stimulus that, when combined with chronic CS exposure, produces enhanced airway inflammation and macrophage activation without causing acute lung injury or mortality. The timing of LPS administration on days 1 and 14 was designed to model recurrent bacterial exacerbations during the course of chronic CS exposure, reflecting the clinical scenario where COPD patients experience intermittent infectious exacerbations superimposed on baseline smoking-induced inflammation.

### Preparation of CS extract (CSE)

Cigarette smoke extract (CSE) was prepared according to a previously described method with minor modifications^16^. Briefly, mainstream cigarette smoke was generated from commercially available filtered cigarettes (11 mg tar, 12 mg CO, 1.0 mg nicotine; Double Happiness, Shanghai Tobacco Group, China). A total of 15 cigarettes were smoked sequentially over a 5-minute period, rather than 5 minutes per cigarette.

Cigarettes were connected to a controlled vacuum-assisted smoking apparatus that generated intermittent smoke draws rather than continuous suction, thereby avoiding excessive combustion temperature associated with uninterrupted vacuum flow. The smoke was bubbled directly into 40 mL of phosphate-buffered saline (PBS) through a connecting tube attached to the cigarette filter.

Following smoke collection, the suspension was filtered through a 0.22 μm membrane to remove large particulates and microbial contaminants. The pH of the resulting solution was measured and adjusted to 7.2–7.4 using sterile NaOH or HCl as required. The absorbance at 320 nm was measured to normalize CSE concentration, which was defined as 100% CSE. Aliquots were stored at -80°C and freshly diluted to the indicated working concentrations prior to each experiment.

### Cell culture and treatment

The Chinese Academy of Sciences Type Culture Bank provided RAW 264.7 cells (Shanghai, China). Cells were grown in DMEM media with 10% fetal bovine serum (Gibco, 10082147), penicillin, and streptomycin. Passaged cells were grown routinely. All experiments were done three times separately. RAW 264.7 cells received 10% CSE for 24 hours after serum fasting.

### Pulmonary function evaluation

Following the final exposure, mice were anesthetized with intraperitoneal pentobarbital sodium (50 mg/kg). Adequate depth of anesthesia was confirmed by the absence of a pedal withdrawal reflex. A midline cervical incision was then made, and the trachea was surgically exposed and cannulated with a sterile metal catheter, which was secured with surgical suture to prevent air leakage.

To eliminate spontaneous respiratory effort and ensure stable and reproducible measurements, mice received a neuromuscular blocking agent to induce diaphragmatic paralysis prior to mechanical ventilation. Animals were then connected to a computer-controlled small-animal ventilator, and pulmonary function was assessed using the AniRes2005 Lung Function System (version 3.5; Bestlab Technology Co., Beijing, China).

Lung function parameters, including forced vital capacity (FVC), forced expiratory volume at 0.05 s (FEV_0.05_), forced expiratory volume at 0.1 s (FEV_0.1_), FEV_0.05_/FVC, FEV_0.1_/FVC, lung resistance (RL), and dynamic lung compliance (Cdyn), were recorded and analyzed according to the manufacturer’s protocol.

### Euthanasia and tissue collection

Upon completion of pulmonary function testing, mice were euthanized under deep anesthesia by exsanguination followed by cervical dislocation, in accordance with institutional animal care guidelines.

Blood was collected via cardiac puncture, allowed to clot at room temperature for 30 minutes, and centrifuged at 3000 rpm for 10 minutes at 4°C to obtain serum. Serum samples were aliquoted and stored at -80°C until analysis.

After euthanasia, bronchoalveolar lavage fluid (BALF) was collected by cannulating the trachea and lavaging the left lung three times with cold phosphate-buffered saline (PBS). BALF supernatants were collected after centrifugation and stored at -80°C for cytokine analysis.

Following BALF collection, lungs were carefully dissected and processed for downstream analyses. The right lung lobes were divided for molecular and cellular assays, including Western blotting, qRT-PCR, and flow cytometry. For flow cytometry, freshly isolated lung tissue was immediately processed into single-cell suspensions as described below.

The left lung was gently inflated with 4% paraformaldehyde at a constant pressure and fixed overnight for histological and immunohistochemical analyses. Fixed lung tissues were then embedded in paraffin, sectioned, and stained as described in the corresponding subsections.

Due to tissue requirements for different assays, not all measurements were performed on the same lung lobes; however, all experimental groups were processed using identical tissue allocation protocols to ensure consistency across analyses.

### Enzyme-linked immunosorbent assay (ELISA)

By using ELISA Assay (Elabscience, Wuhan, China), we evaluated the amounts of IL-1β, TNF-α, IL-6, and IL-10 in serum BALF and supernatants from RAW 264.7 cells.

### Flow cytometry

Freshly collected lung tissue was minced and digested with collagenase I (BioFroxx, Einhausen, Germany) and DNase (Solarbio, Beijing, China) in a water bath at 37^o^C for 2 hours. Cell suspensions were passed through a 40 μm cell strainer and centrifuged to obtain a single-cell suspension. Cells were stained with the following fluorophore-conjugated antibodies: CD45-Perc-cy5.5 (BD Biosciences, 561869), F4/80-APC (BioLegend, 123115), CD11b-BB515 (BD Biosciences, 564454), CD86-BV421 (BioLegend, 105013), L/D-APC-CY7 (BD Biosciences, 565321), and CD206-PE (Thermo Fisher Scientific, 12-2061-82). For RAW264.7 cell analysis, cells were detached with 0.25% trypsin; centrifuged for 5 minutes to remove supernatant, and washed twice in PBS. The cells were then treated in the dark for 30 minutes with the primary antibodies CD86-BV421 (BioLegend, 105013) and CD206-PE (Thermofisher, 141706). After incubation, the cells were washed and resuspended in PBS that contained 2% fetal bovine serum. The final step was the resuspension of the cells in FACS staining solution and the acquisition of data using an LSRFortessa flow cytometer (BD Biosciences). FlowJo version 10.8.1 (FlowJo LLC, Ashland, OR, USA) was utilized for data analysis and visualization^15^.

### H&E staining

To investigate histopathological alterations in lung tissues, hematoxylin and eosin (H&E) staining was performed. Lung tissues were fixed overnight with 4% formaldehyde in phosphate buffer, dehydrated, embedded in paraffin, and cut into 4 μm thick sections. Sections were stained with hematoxylin and eosin (Solarbio G1120, China) according to the manufacturer’s instructions. A photograph documentation facility inspected the stained slides under a light microscope after staining (Olympus, Tokyo, Japan). All histology exams were conducted blindly by two individuals. The lesion area in the pathological sections was measured with Image-Pro Plus, and the pathological score was evaluated in accordance with the standards^15^.

### Immunohistochemistry for iNOS

Immunohistochemistry was utilized to examine the level of iNOS in lung tissue. Briefly, paraffin-embedded lung tissue slices were deparaffinized and rehydrated before antigen retrieval for 20 minutes; 3% hydrogen peroxide inhibited the activity of endogenous peroxidase. The slides were blocked with 5% BSA and kept overnight with the primary antibody at 4°C. After that, the slices were developed with a 3–3'-diaminobenzidine solution after being incubated with the appropriate HRP polymers. When the hematoxylin counterstaining was completed, the slides were allowed to dry completely before being mounted using a mounting medium.

Fractions of the mean optical densities of iNOS immunostaining in lungs from mice analyzed using Image-Pro Plus software (Media Cybernetics, USA). Five random non-overlapping fields were analyzed at the same magnification and exposure settings for each animal. The MOD (mean optical density) for each mouse was calculated by dividing the integrated optical density by the area of the respective tissue. Group averages (mouse averages) of the MOD values are used for statistical evaluation with blinding on the day the data are analyzed. The animals were analyzed as n=3–5 mice in each group, and five microscopy fields were analyzed for each animal for statistical comparison.

### Western blotting

Using Western blotting, the protein expression levels of each group were analyzed for alterations. RIPA lysis buffer was used in the process of protein extraction from either cells or tissue samples (P0013B, Beyotime, Beijing, China), and the protein’s quality was detected by using the BCA technique. Using 10% sodium sulfate polyacrylamide gel electrophoresis (SDS-PAGE), normalized protein samples were loaded and transferred to PVDF membranes. INOS (18985-1-AP, Cell Signaling Technology, Proteintech, 1:1000), TNF-α(60291-1-Ig, Cell Signaling Technology, Proteintech, 1:2000), IL-1β(16806-1-AP, Cell Signaling Technology, Proteintech, 1:1000), IL-6 (21865-1-AP, Cell Signaling Technology, Proteintech, 1:1000), IL-10 (60269-1-Ig, Cell Signaling Technology, Proteintech, 1:5000), p-NF-κB/p-p65 (3039s, Cell Signaling Technology, 1:500), p65 (10745-1-AP, Cell Signaling Technology, Proteintech, 1:500), NLRP3 (19771-1-AP, Cell Signaling Technology, Proteintech, 1:1000) and ARG-1 (Thermo Fisher Scientific, NY, USA). The membranes were then treated at room temperature with secondary antibodies. The target protein’s expression was counted using the Bio-Rad imaging system.

All experiments have been done in exactly the same quantity of protein per group; all protocols used equal numbers of samples in their analyses, and so the same sample was used across all analytical methods, including the same membrane used for multiple target proteins and the β-actin that had been identified on these membranes (to prove the quantity of total protein present). Therefore, membranes that were identified with β-actin will be reused as a loading control for future target proteins. To aid reader comprehension and increase transparency, full-length uncropped images of the β-actin will be shown as supplementary figures with the associated target protein images (Supplementary file Figures 3 and 4).

### qRT-PCR assay

Using TRIzol reagent, total RNA was isolated from cells. Using Nanodrop, the quality and amount of the RNA samples were analyzed (Thermo Scientific 2000c, USA). A reverse transcription kit was used to reverse-transcribe whole RNA (RR047A, Takara, Japan). The qRT-PCR operations were carried out. Normalization of GAPDH relative to the control was used to calculate the Ct method and relative changes in mRNA levels. The employed primers are given in Supplementary file Table 1. The data were evaluated in triplicate using Bio-Rad CFX 2.1 Manager software version 2.1 (Bio-Rad Laboratories, Hercules, CA, USA).

### Statistical analysis

The data was analyzed using GraphPad Prism version 8.0 (GraphPad Software, San Diego, CA, USA)^12^. The results are reported as mean ± SD values. Student’s unpaired t-test and one-way ANOVA, followed by the Newman-Keuls post-test if statistically significant (p<0.05), were used to analyze the differences between groups. The following p-values indicate statistical significance: *p<0.05; **p<0.01; ***p<0.001, ****p<0.0001. Bar graphs were used to summarize group-level differences for clarity, as the primary objective was to compare mean responses between experimental groups; individual sample sizes (n) and measures of variability are explicitly reported to ensure transparency.

## RESULTS

### The pulmonary function and pathological morphological changes in the lung tissue of CS-exposed mice

**Figure 1:**
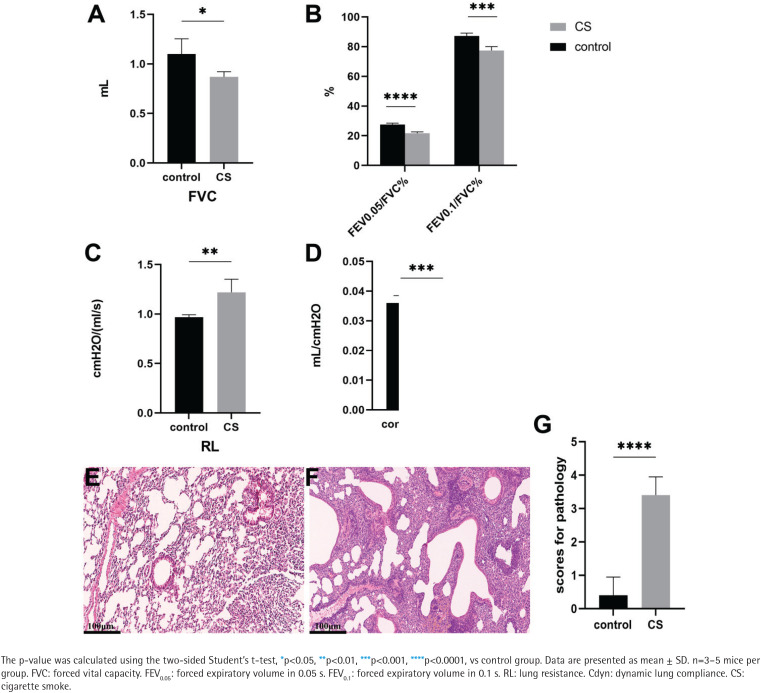
Lung function and hematoxylin and eosin (H&E) staining of air-exposed mice (control) and CS-exposed (CS) mice. Histological images are representative of lung sections from each group: A-D) Pulmonary function was shown as FVC (A), FEV_0.05_/FVC and FEV_0.1_/FVC (B), RL (C), and Cdyn (D) of all mice; E-F) The pathological changes in lung tissues were observed by H&E staining for the control group (E) and CS group (F) (Scale bars, 100 μm); G) Histograms showing the pathological scores for the different groups

Functional impairment caused by exposure to cigarette smoke (CS) was determined by pulmonary function, assessing forced vital capacity (FVC), forced expiratory volume (FEV), dynamic lung compliance (Cdyn), and lung resistance (RL). When compared to control mice exposed to air, CS-exposed mice showed significantly lower FVC (0.87 ± 0.05 vs 1.10 ± 0.15 mL in controls, p<0.05; Figure 1A), reduced FEV_0.05_/FVC (21.70 ± 0.96 vs 27.50 ± 0.94%, p<0.0001) and FEV_0.1_/FVC ratios (77.39 ± 2.62 vs 87.21 ± 1.95%, p<0.001; Figure 1B), and decreased Cdyn (0.027 ± 0.003 vs 0.036 ± 0.003 mL/cmH_2_O, p<0.001; Figure 1D). Conversely, RL was significantly increased in CS-exposed mice compared to controls mice (1.22 ± 0.13 vs 0.97 ± 0.02 cmH_2_O/(mL/s), p<0.01; Figure 1C), indicating that CS exposure has a detrimental effect on pulmonary function consistent with obstructive airway disease.

Histological analyses of lung tissue using hematoxylin and eosin (H&E) staining demonstrated significant pulmonary inflammatory changes in the lungs of CS-exposed mice compared with controls. CS-exposed mice showed marked infiltration of inflammatory cells (predominantly macrophages and neutrophils), increased alveolar wall thickness (22.4 ± 3.6 vs 12.8 ± 2.1 μm in controls, p<0.001), and disruption of the normal alveolar architecture (Figures 1E and 1F). Pathology scores, based on a semiquantitative assessment of lung injury (0–12 scale), were significantly higher in CS-exposed mice than in control mice (3.40 ± 0.55 vs 0.40 ± 0.55, p<0.0001; Figure 1G).

The histological images illustrated represent selected fields that were used for pathology scoring but do not depict the total extent of tissue injury, airway wall remodeling, or emphysematous enlargement of lung air space. No appreciable enlargement of the diameter of alveolar air space was observed in this study under the experimental conditions utilized.

### Levels of cytokines and factors in BALF and serum of CS-exposed mice

ELISA was used to evaluate the level of M1 pro-inflammatory and M2 anti-inflammatory cytokines in BALF and serum. In BALF of CS-exposed mice (Supplementary file Figure 5A), there was a significant increase in TNF-α (290.6 ± 7.58 vs 125.6 ± 5.30 pg/mL in controls, p<0.0001), IL-6 (67.92 ± 3.02 vs 44.42 ± 2.62 pg/mL in controls, p<0.001), and IL-1β (120.3 ± 8.24 vs 87.52 ± 3.40 pg/mL, p<0.01), while IL-10 was markedly decreased (46.12 ± 1.71 vs 77.19 ± 3.96 pg/mL, p<0.001). Similar patterns were observed in serum (Supplementary file Figure 5B): TNF-α (208.1 ± 13.51 vs 84.99 ± 11.35 pg/mL, p<0.001), IL-6 (44.87 ± 2.85 vs 24.85 ± 2.46 pg/mL, p<0.001), IL-1β (28.76 ± 2.75 vs 16.36 ± 1.72 pg/mL, p<0.01), and IL-10 (37.29 ± 4.64 vs 50.68 ± 5.80 pg/mL, p<0.05). These results indicate that increased M1 pro-inflammatory cytokines and reduced M2 anti-inflammatory cytokines contribute to the exacerbated inflammatory environment following CS exposure.

### CS exposure markedly increases iNOS expression in lung macrophages

Immunohistochemical analysis revealed markedly increased expression of the M1 macrophage marker inducible nitric oxide synthase (iNOS) in the lung tissue of CS-exposed mice versus control (Supplementary file Figures 1A and 1B). Quantitative analysis of the mean optical density (MOD) of iNOS-positive staining demonstrated a significant increase in the CS group (0.166 ± 0.028) compared to controls (0.047 ± 0.016, p<0.0001; Supplementary file Figure 1C), indicating that CS exposure induces pro-inflammatory macrophage activation in lung tissue.

### CS exposure induces M1-biased macrophage polarization in mouse lung tissue

We used western blotting and qPCR to analyze the expression of M1- and M2-associated markers in mouse lung tissue. Western blot analysis (Supplementary file Figure 1D) demonstrated a significant increase in M1-associated proteins in CS-exposed mice compared to controls: IL-1β (2.9-fold increase, p<0.01), INOS (2.8-fold increase, p<0.01), TNF-α (3.2-fold increase, p<0.001), and IL-6 (3.5-fold increase, p<0.001). Conversely, M2-associated markers were significantly decreased: ARG-1 (62.3% reduction, p<0.01) and IL-10 (58.7% reduction, p<0.01), compared to the lung tissue of mice in the control group.

qRT-PCR confirmed these findings at the mRNA level (Supplementary file Figures 1E–1M). M1-associated gene expression was significantly upregulated in CS-exposed mice: INOS (2.15 ± 0.47 vs 1.00 ± 0.17 in controls, p<0.05; Supplementary file Figure 1E), CD86 (1.50 ± 0.24 vs 0.99 ± 0.09, p<0.05; Supplementary file Figure 1F), TNF-α (1.66 ± 0.24 vs 0.10 ± 0.02, p<0.01; Supplementary file Figure 1G), IL-1β (2.90 ± 0.18 vs 1.01 ± 0.09, p<0.0001; Supplementary file Figure 1H), and IL-6 (2.24 ± 0.14 vs 0.94 ± 0.11, p<0.001; Supplementary file Figures 1I and 1J). In contrast, M2-associated gene expression was significantly reduced: ARG-1 (0.75 ± 0.02 vs 1.01 ± 0.12, p<0.05; Supplementary file Figures 1K and 1L) and IL-10 (0.60 ± 0.03 vs 0.99 ± 0.04, p<0.001; Supplementary file Figure 1M). No significant difference was observed in CD206 mRNA expression between groups (0.85 ± 0.11 vs 0.97 ± 0.09, p>0.05; Supplementary file Figure 1L).

It should be noted that the increased expression of M1-associated markers detected by Western blotting and qRT-PCR reflects enhanced inflammatory activation at the molecular level, whereas flow cytometric analysis provides a population-based assessment of macrophage phenotypes. These readouts capture complementary but not identical aspects of macrophage polarization. Given the high plasticity and dynamic nature of macrophage phenotypes, upregulation of M1-related genes and proteins may occur without a proportional expansion of M2 macrophage populations, resulting in an apparent reduction in the M2/M1 ratio. Thus, the molecular and population-level data together indicate a shift toward M1-biased inflammatory activation rather than a static binary conversion between M1 and M2 states.

### The effect of CSE on the polarization of macrophages in RAW 264.7 cells

To characterize macrophage polarization under sustained inflammatory stimulation, we first quantified representative pro- and anti-inflammatory cytokines in CSE-treated RAW264.7 cells by ELISA. As shown in Supplementary file Figure 5C, 10% CSE for 24 hours exposure significantly increased the secretion of TNF-α (1.94 ± 0.03 vs 1.51 ± 0.10 pg/mL in controls, p<0.05), IL-6 (0.45 ± 0.002 vs 0.27 ± 0.009 pg/mL, p<0.0001), and IL-1β (0.12 ± 0.00 vs 0.10 ± 0.00 pg/mL, p<0.01), while reducing IL-10 levels (0.15 ± 0.00 vs 0.17 ± 0.00 pg/mL, p<0.001) compared with controls, indicating a pro-inflammatory milieu consistent with M1-biased polarization.

Western blot analysis (Supplementary file Figure 2A) revealed dose-dependent increases in M1-associated proteins with increasing CSE concentrations (5%, 10%). At 10% CSE, significant increases were observed: TNF-α (3.4-fold increase vs control, p<0.01), INOS (3.8-fold increase, p<0.001), IL-1β (3.2-fold increase, p<0.01), and IL-6 (4.1-fold increase, p<0.001). Conversely, M2-associated markers showed significant dose-dependent decreases: ARG-1 (58.4% reduction at 10% CSE, p<0.01) and IL-10 (64.2% reduction at 10% CSE, p<0.01).

qRT-PCR analysis confirmed these findings at the mRNA level (Supplementary file Figures 2B–2I). At 10% CSE, M1-associated gene expression was significantly upregulated: INOS (10% CSE: 5.35 ± 0.48; 5% CSE: 2.39 ± 0.12; .control: 1.01 ±0.14, p<0.0001; Supplementary file Figure 2B), CD86 (10% CSE: 7.86 ± 0.84; 5% CSE: 1.73 ± 0.13; .control: 1.01 ± 0.19, p<0.0001; Supplementary file Figure 2C), TNF-α (10% CSE: 9.67 ± 0.71; 5% CSE: 3.53 ± 0.08; .control: 1.02 ± 0.25, p<0.0001; Supplementary file Figure 2D), IL-1β (10% CSE: 4.45 ± 0.59; 5% CSE: 1.77 ± 0.06; .control: 1.00 ±0.02, p<0.0001; Supplementary file Figure 2E), and IL-6 (10% CSE: 4.47 ± 0.43; 5% CSE: 1.81 ± 0.16; .control: 1.01 ± 0.19, p<0.0001; Supplementary file Figure 2F). M2-associated gene expression was significantly reduced: ARG-1 (10% CSE: 0.72 ± 0.12; 5% CSE: 0.91 ± 0.03; .control: 1.00 ± 0.04, p<0.05; Supplementary file Figure 2G) and IL-10 (10% CSE: 0.71 ± 0.06; 5% CSE: 1.11 ± 0.15; .control: 1.00 ± 0.05, p<0.01; Supplementary file Figure 2I). No significant difference was observed in CD206 expression (10% CSE: 0.78 ± 0.04; 5% CSE: 0.85 ± 0.12; .control: 1.02 ± 0.23, p>0.05; Supplementary file Figure 2H).

### CS and CSE exposure increase the proportion of M1 macrophages without significantly expanding M2 populations

**Figure 2:**
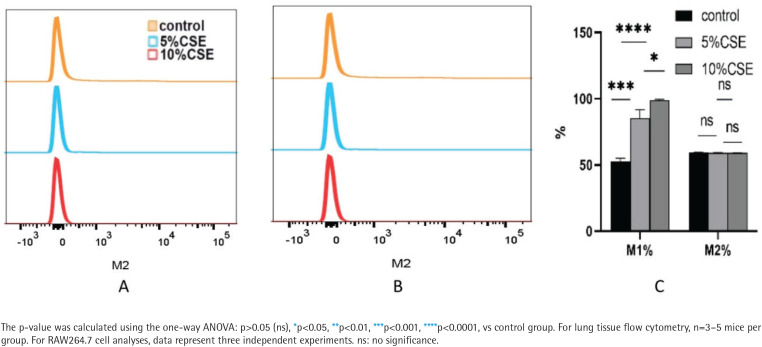
Flow cytometric analysis of macrophage surface marker expression in lung tissues of mice and RAW264.7 cells. Representative FACS plots of CD86^+^ (F4/80^+^CD86^+^) (A) and CD206^+^ (F4/80^+^CD206^+^) (B) macrophage populations in RAW264.7 cells following cigarette smoke extract (CSE) treatment. Quantitative analysis of CD86^+^ and CD206^+^ RAW264.7 cells is shown in (C). CD86 and CD206 were used as representative surface markers associated with pro-inflammatory and alternatively activated macrophage-associated profiles, respectively, and do not alone define fixed macrophage phenotypes

Flow cytometry was used to assess the proportion of M1-like (CD86^+^) and M2-like (CD206^+^) macrophages in lung tissue and RAW264.7 cells. In mouse lung tissue (Figure 2; and Supplementary file Figures 6 and 7), CS-exposed mice showed a significantly higher percentage of CD86^+^ macrophages within the total F4/80^+^ macrophage population compared to controls (7.13 ± 1.78% vs 3.28 ± 0.52%, p<0.01; Supplementary file Figure 7D). In contrast, the percentage of CD206^+^ macrophages was not significantly different between groups (3.84 ± 0.66% vs 2.93 ± 0.43%, p>0.05; Supplementary file Figure 7D). Consequently, the CD206^+^/CD86^+^ ratio was dramatically reduced in CS-exposed mice (0.55 ± 0.07 vs 0.90 ± 0.05, p<0.001; Supplementary file Figure 7C), indicating a shift toward M1-biased polarization.

*In vitro* analysis of RAW264.7 cells treated with 10% CSE for 24 hours (Supplementary file Figures 6F–6H) corroborated these findings. CSE treatment significantly increased the proportion of CD86+ RAW264.7 cells compared to controls (10% CSE: 98.87 ± 0.70; 5% CSE: 85.37 ± 6.35; .control: 52.87 ± 2.30, p<0.0001). These data provide consistent evidence that CS and CSE exposure preferentially induce M1-like macrophage polarization without significantly expanding M2-like populations.

### CS and CSE promote M1 macrophage polarization by regulating NF-κB/NLRP3 signaling pathway

To investigate the molecular mechanisms underlying CS-induced M1 polarization, we examined the NF-κB/NLRP3 signaling pathway (Supplementary file Figure 1). Western blot analysis (Supplementary file Figure 1D) revealed significantly increased phosphorylation of P65, p-P65 in lung tissues of CS-exposed mice compared to controls (3.6-fold increase, p<0.01), while total p65 expression remained unchanged (1.1-fold change, p>0.05). NLRP3 protein expression was also significantly elevated in CS-exposed mice (3.2-fold increase, p<0.01). qRT-PCR analysis confirmed these findings at the mRNA level (Supplementary file Figures 1I–1N), showing increased expression of NLRP3 (2.43 ± 0.26 vs 1.01 ± 0.05, p<0.001) and NF-κB1 (2.02 ± 0.11 vs 1.02 ± 0.08, p<0.001) in CS-exposed mice (Supplementary file Figures 1I and 1N).

In RAW264.7 cells, CSE stimulation produced dose-dependent increases in p-p65 and NLRP3 protein expression (Supplementary file Figure 2). At 10% CSE, p-p65 was increased 4.2-fold (p<0.01) and NLRP3 was increased 3.9-fold (p<0.01) compared to controls, with no significant change in total p65. qRT-PCR analysis (Supplementary file Figures 2J and 2K) demonstrated significant upregulation of NLRP3 mRNA (10% CSE: 7.98 ± 0.56; 5% CSE: 3.91 ± 0.56; .control: 1.01 ± 0.16, p<0.0001) and NF-κB1 mRNA (10% CSE: 4.70 ± 0.36; 5% CSE: 2.84 ± 0.20; .control: 1.00 ±0.09, p<0.0001) at 10% CSE. These results suggest that CS and CSE exposure promote M1 macrophage polarization through activation of the NF-κB/NLRP3 signaling pathway.

## DISCUSSION

The current research provides evidence that a combined exposure to cigarette smoke and lipopolysaccharide results in significant M1-biased macrophage polarization in both human and animal models of COPD. Significant findings from this study include: 1) Mice exposed to cigarette smoke had impaired lung function, as evidenced by decreased FEV_0.05_/FVC (21.70 ± 0.96 vs 27.50 ± 0.94%, p<0.0001) and increased lung resistance (1.22 ± 0.13 vs 0.97 ± 0.02 cmH_2_O/(mL/s), p<0.01; Figure 1C) The production of multiple pro-inflammatory cytokines (TNF-α, IL-6 and IL-1β) was significantly increased, while the production of the anti-inflammatory cytokine IL-10 was significantly decreased in both animal and human models; 3) M1-associated markers (iNOS, CD86) were both increased at both the protein and mRNA levels, while M2-associated markers (ARG-1, IL-10) were both decreased; 4) The flow cytometry analysis confirmed that the proportion of CD86+ M1 macrophages was significantly increased in cigarette smoke-treated mice versus controls (7.13 ± 1.78% vs 3.28 ± 0.52%, p<0.01) and that the proportion of CD206^+^ M2 macrophages was not significantly different between the two groups; and 5) The observed phenotypic changes were associated with a significant increase in the activation of the NF-κB/NLRP3 signaling pathway, as evidenced by an increase in p-p65 and NLRP3 expression. Taken together, the experimental data from this study show that cigarette smoke constituents directly promote pro-inflammatory macrophage activation, and therefore provide further support for the ability of cigarette smoke to induce M1-polarized macrophages.

One of the most common adult chronic respiratory diseases worldwide is COPD, which is characterized by long-term damage to the lungs resulting in persistent inflammation and irreversible airway obstruction due to a lack of airflow. Cigarette smoke (CS) is the most commonly cited risk factor for COPD^17^. Macrophages play an important role in regulating pulmonary inflammation, and macrophages remove cellular debris, monitor for foreign material, and regulate inflammatory responses^18^. Macrophage phenotype and function are altered through the macrophage’s ability to adapt to the local microenvironment; this phenomenon is called macrophage plasticity, also referred to as macrophage polarization^19^. The relationship between cigarette smoke-induced macrophage activation and airway inflammation in the development of COPD has been well established; however, there is still uncertainty surrounding the predominance of pro-inflammatory versus alternatively activated macrophage-associated phenotypes in COPD^18,20^.

Cigarette smoke-only exposure models are frequently employed to determine the underlying mechanisms of COPD; however, they do not accurately reflect the complex pattern of inflammation present in patients experiencing an infectious exacerbation^12^. In the present experiment, we used a combined cigarette smoke–lipopolysaccharide (LPS) exposure model to create a clinically relevant inflammatory microenvironment typically seen in chronic cigarette smokers (i.e. chronic exposure to cigarette smoke) and with superimposed exposure to bacterial-derived stimuli^21,22^. This model is not intended to be a substitute for traditional cigarette smoke-only exposure models, but rather provide a more accurate description of an exaggerated inflammatory phenotype similar to that of COPD patients with bacterial infection^23^. Therefore, macrophage responses resulting from this study should be interpreted in the context of compounded inflammatory stress.

Based on previous studies^24,25^, M1 pro-inflammatory macrophages are elevated in COPD patients compared to non-COPD controls. The M1 macrophage type and increased level of iNOS expression were previously reported in alveolar macrophages of COPD patients due to exposure to various chemicals present in the cigarette extract^26^. The presence of elevated M1 pro-inflammatory cytokines has been associated with chronic inflammation, emphysema, and mucus production in laboratory assays for COPD patients. According to Feng et al.^27^, cigarette smoke exposure can cause M1 macrophage polarization. In addition, Wang et al.^28^ found that CSE-treated airway epithelial cell-derived exosomes cause M1 macrophage polarization and enhance CS-induced lung damage and dysfunction.

The role of macrophage diversity in COPD is still unclear as well as the contradictory data between previous studies to each other and this study. Some reports indicate that there are more M2 macrophage phenotypes in the bronchoalveolar lavage fluid (BALF) of COPD compared with controls and that they have increased levels of IL-4, IL-13, IL-10, smoke exposure induces M2 macrophage polarization and they produce matrix metalloproteinase-12 (MMP-12)^11,12,29^. MMP-12 is derived from IL-4-stimulated M2 macrophages, and has a role in the development of emphysema^30-32^. Furthermore, Beyers et al.^33^ demonstrated that IL-13 induces M2 macrophage polarization and mucin gene expression in COPD mouse models and humans. In contrast, several studies show a dramatic increase in the co-expression of all four M1 and M2 macrophage and macrophage non-polarization in COPD^34,35^. Bazzan et al.^34^ investigated the alveolar macrophage polarization profile in a range of subjects from healthy to very severe COPD and found that total alveolar macrophages exhibited both M1 and M2 characteristics, and dual polarization was observed throughout the course of the disease. Therefore, the state and function of macrophages, their polarized state(s) and their integration into the pathogenesis of COPD is very complex.

A key aspect of this work is the combination of *in vivo* and *in vitro* methods to examine macrophage polarization and associated inflammatory changes related to chronic obstructive pulmonary disease (COPD) caused by cigarette smoke exposure (CS) exposure in an animal model of combining CS and lipopolysaccharide (LPS) administration. The *in vivo* CS+LPS exposure model closely resembles the natural complex microenvironment present in human lung tissue, where macrophage interactions occur with epithelial cells, fibroblast cells, and local recruited inflammatory cells. The animal model captures normal complexities associated with chronic exposure duration (e.g. one-time to extend) and systemic effects. The animal data showed significant differences between CS+LPS exposed and CS-exposed groups with respect to pathological manifestations (inflammatory cell infiltration, alveolar wall thickness: 22.4 ± 3.6 vs 12.8 ± 2.1 μm, p<0.001, inflammatory pathway score: 3.40 ± 0.55 vs 0.40 ± 0.55, p<0.0001; Figure 1G), demonstrating that the CS+LPS animal model exhibits many of the pathological manifestations observed in COPD.

On the other hand, the *in vitro* study investigated RAW264.7 macrophages using CSE exposure, where experimental exposure parameters, including CSE concentration and duration of exposure, could be controlled. The availability of defined experimental conditions for examining RAW264.7 cells allowed for dose-response analysis. In addition, the independent exposure of RAW264.7 cells to CSE and separation of the macrophage subset from multicellular interaction effects allowed for determination of the mechanisms by which components in cigarette smoke could directly change macrophage phenotype. Specifically, the data demonstrate a dose-dependent increase in M1 markers (e.g. iNOS, CD86, TNF-α, IL-1β, IL-6) and a corresponding decrease in M2 markers (ARG-1, IL-10) in RAW264.7 cells with increasing CSE concentrations (5%, 10%), indicating that constituents in cigarette smoke are capable of directly programming macrophages to a pro-inflammatory phenotype.

The concordance of results from both *in vivo* and *in vitro* studies, specifically the high percentages of M1-biased polarized macrophages (7.13 ± 1.78% vs 3.28 ± 0.52%, p<0.01 CD86+ macrophages *in vivo*, 10% CSE: 98.87 ± 0.70; 5% CSE: 85.37 ± 6.35; .control: 52.87 ± 2.30, p<0.0001 *in vitro*) and NF-κB/NLRP3 activation (3.6× vs 4.2× p-p65 *in vivo*/*in vitro*), suggest that components of cigarette smoke directly program macrophages towards a pro-inflammatory phenotype independent of other cell types. However, because the magnitude of inflammatory responses is generally larger *in vivo* than *in vitro* due to the amplifying effects of an inflammatory milieu and cellular crosstalk are present in lung tissue, this combined approach to studying inflammation provides additional support for the findings of both models and lends further support to the validity of RAW264.7 cells for mechanistic studies of CS-induced macrophage polarization while recognizing that primary alveolar macrophages would add additional translational relevance.

In addition, this research demonstrated how the molecular pathways involved in CS or CSE induce macrophage polarization towards an M1 phenotype. First, we studied whether or not CS or CSE caused changes in the expression levels of p-NF-κB/p-p65 and NLRP3 in the lungs of CS mice, which was determined to be significantly elevated from baseline in both the lung and in isolated macrophages from mice exposed to CS. The expression levels of p-NF-κB and NLRP3 were found to be at 3.6× or 3.2× greater than baseline levels for CS mice and 4.2× or 3.9× greater for RAW264.7 macrophages activated with CSE (10% CSE), respectively. Based upon these results there appears to be an association between NF-κB/NLRP3 signaling pathways and macrophage polarization, consistent with some previous studies supporting the association of NF-κB with disease pathology. As a result of this evidence, we conclude that NLRP3 is a mediator of macrophage activation and differentiation through the NF-κB/NLRP3 pathways^32^. Along these lines, NLRP3-mediated activation of macrophages is a key component for tissue repair and healing^6^. Experiments have shown that M1 phenotype macrophages stimulated with LPS became M2 phenotype macrophages following inhibition of NLRP3^13^. Similarly, the findings of Wen et al.^28^ confirmed that NLRP3 and NF-κB signaling pathways play a role in the polarization of M1 macrophages.

By using various experimental techniques to create a unified model for the analysis of macrophage polarization status, cytokine production, and signaling pathway activation, this research demonstrates a new understanding of the role of macrophage M1 phenotypes in relation to inflammation associated with chronic obstructive pulmonary disease (COPD). Results indicate that, during prolonged periods of inflammasome activation, there is an increase in M1 polarization, suggesting that continued activation of this pathway may be implicated in the development of COPD as evidenced by shifts in the balance between M1 and M2 macrophages.

### Limitations

This study has several limitations that should be acknowledged when interpreting the findings. Firstly, differential clinical assessments within bronchoalveolar lavage fluid (BALF) for inflammatory cell counts and cytospin characterization of inflammatory cells were not performed. Neutrophil infiltration, activation, apoptosis, and efferocytosis – known influencers in COPD pathogenesis – were not measured directly. The study did not assess the presence of potential neutrophil – derived chemotactic signals that may have impacted macrophage activation.

Second, the experimental design failed to incorporate key control groups (CS only and LPS only), making it impossible to separate the contribution from each experimental stimulus onto macrophages from both the respective stimuli and from CS and LPS combined. Therefore, we cannot conclude that the observed M1 polarization was primarily due to the combination of CS and LPS, rather than due only to their separate stimulation.

Third, macrophage accumulation within the lung tissues was primarily assessed through functional and molecular analyses, rather than histological staining of macrophages, which limits the ability to define precise spatial location of macrophages within the lung compartments.

Fourth, the smaller sample size (n=5/group) limits statistical power to detect smaller group differences, increasing potential for type II error in some comparisons. Fifth, the findings of this study show an association but not causation between activation of NF-kB or NLRP3 and M1 polarization. As we did not perform mechanistic inhibition experiments (e.g. using NF-kB inhibitors like BAY 11-7082 and NLRP3 inhibitors like MCC950), we cannot say that signaling through NF-kB and NLRP3 causes M1 polarization secondary to cigarette smoke (CS) or simply correlates with M1 polarization after exposure to CS.

Sixth, using RAW264.7 cells instead of primary alveolar macrophages may limit the ability to translate the results because immortalized cell lines typically behave differently than primary cells. Conducting future studies on primary human alveolar macrophages obtained from patients with chronic obstructive pulmonary disease (COPD) would help improve the clinical relevance of the results. Seventh, the histological assessments were based on representative tissue sections and semi-quantitative scores without conducting direct morphometric measurements of airway wall thickness and alveolar destruction. Future studies that include systematic morphometric analyses would provide detailed evaluation of structural remodeling occurring within COPD. Eighth, an exaggerated inflammatory phenotype was observed when CS and LPS were used in the exposure model, compared to the stable phenotype observed in people with COPD. These results are not representative of individuals with stable, baseline COPD but rather of individuals with COPD who have a bacterial infection or who are having an acute exacerbation. Ninth, there was no dynamic tracking of the conversion of M1 to M2 or experiments related to the reversal of polarization and no complete marker analysis using more than just CD86/CD206 and selected cytokines was performed. Macrophage polarization occurs in a highly dynamic manner, thus our measurements at a single time point could miss many of the phenotypic shifts occurring in response to chronic inflammation. Tenth, when utilizing 10% CSE for in vitro modeling, the stimulus that results from the exposure used is quite high and, therefore, it does not replicate the chronic exposure that occurs at lower levels with people that smoke cigarettes. Additional dose-response studies with prolonged exposure times at lower doses would be a much more applicable way to replicate chronic cigarette smoke exposure in people with COPD. Finally, since there are no live bacterial challenges performed, we cannot directly compare the effects of polarization of macrophages on host responses to infection. Future studies utilizing live bacterial challenges in mice exposed to CS would provide clinically applicable information regarding the effects of macrophage function during acute exacerbation of COPD.

### Future directions

Because macrophage polarization is very complex and changes when COPD progresses, future studies will focus on these points: 1) longitudinal studies observing macrophage phenotype changes (polarization) during the progress of COPD; 2) Mechanistic studies utilizing pathway inhibitors to demonstrate the causal relationship of NF-kB/NLRP3 activation and M1 polarization; 3) Comparative studies of primary human alveolar macrophages from COPD patients with varying disease stages; 4) Investigate whether reversing macrophage polarization can serve as a potential therapy; 5) Live bacterial challenges to model infectious exacerbations; and 6) Development of more advanced *in vitro* models (e.g. using co-culture systems and lung organoids) that mimic the multiple environments found in the lung.

## CONCLUSIONS

Our results demonstrate that combined cigarette smoke and bacterial inflammatory stimulation elicits a strong, M1-biased macrophage response via activation of NF-kB/NLRP3 pathways. These findings provide insight into the mechanistic basis for macrophage dysregulation during COPD-associated infections and exacerbations. The concordance between in vivo and in vitro models strengthens the evidence that cigarette smoke components directly contribute to pro-inflammatory macrophage polarization. However, since macrophage polarization is extremely dynamic process influenced by the continuously changing lung microenvironment through interactions with external microbes and environmental agents, more research is required to better understand the mechanisms of macrophage polarization and their implications for both basic science and clinical applications of COPD. Future studies should focus on establishing causal relationships through mechanistic inhibition experiments and exploring the therapeutic potential of modulating macrophage polarization in COPD.

## Supplementary Material



## Data Availability

The datasets obtained and analyzed for this study are available from the corresponding author on reasonable request.
