# Supplementary material for: Observation of inflammation and macrophage polarization in an experimental model of cigarette smoke-exposed mice and cigarette smoke extract (CSE)-induced RAW264.7 cells: An experimental preclinical study
Source: Tob Induc Dis. 2026 Jul 26;24:10.18332/tid/221091. doi: 10.18332/tid/221091 (PMC13417965; doi:10.18332/tid/221091)
Supplement: Supplementary file 1 [file TID-24-123-s1.pdf]

Tobacco Induced Diseases

Supplementary file

© 2026 Zhao J. et al.

DOI:
10.18332/tid/221091

The content has been provided by the author(s) and has not been reviewed, verified, or endorsed by European Publishing. It may not have undergone peer review. The views, opinions, and recommendations expressed are solely those of the author(s) and do not necessarily reflect the position of European Publishing. European Publishing accepts no responsibility or liability for any consequences arising from the use of, or reliance on, this content.

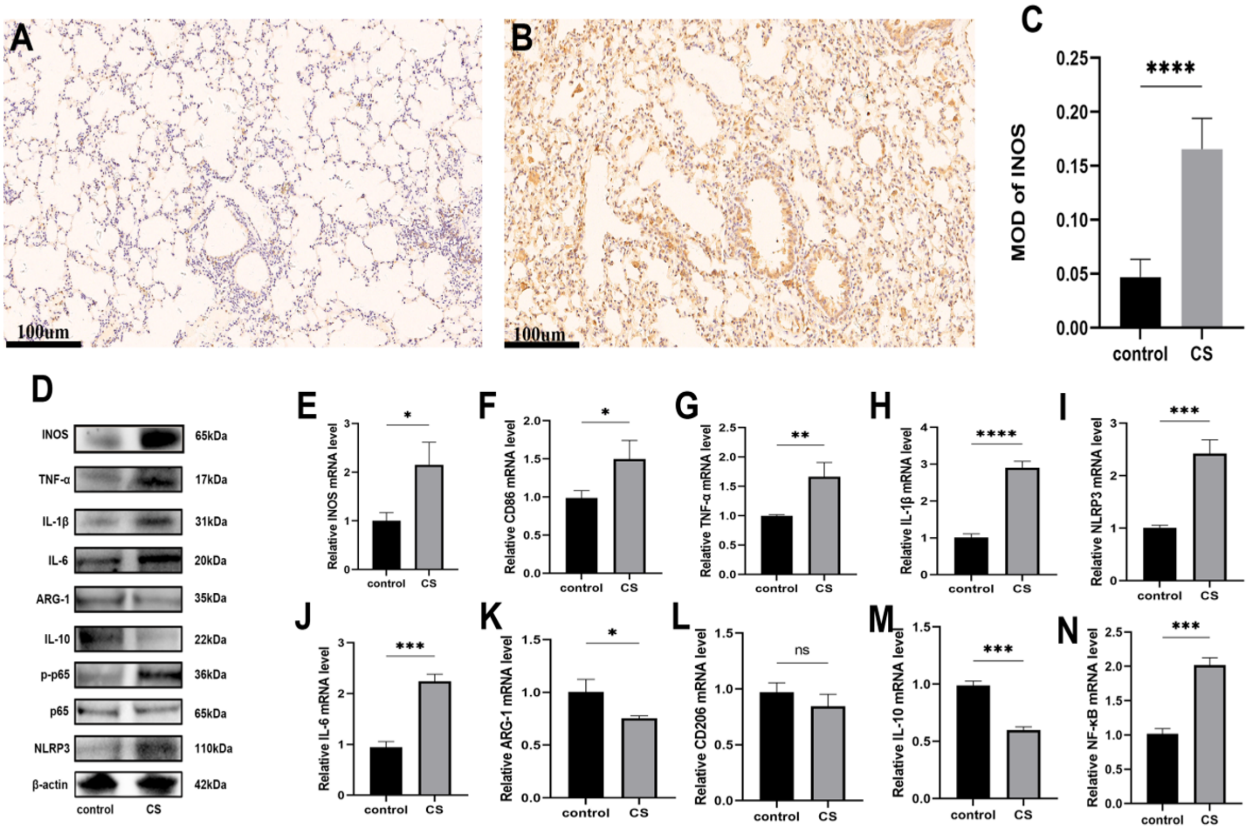

Supplementary Figure S1. Effect and mechanism analysis of lung tissue inflammation and macrophage polarization in air-exposed mice (control) and CS-exposed (CS) mice. Immunohistochemistry analysis for INOS in mice for the control group (A) and CS group (B). The corresponding histogram is shown as (C). Western blots analysis of INOS, TNF-α, IL-1β, IL-6, ARG-1, IL-10, p-p65, p65 and NLRP3 in mice (D). The mRNA expression of iNOS (E), CD86 (F), TNF-α (G), IL-1β(H), NLRP3 (I), IL-6 (J), ARG-1 (K), CD206 (L), IL-10 (M) and NF-κB (N) in mice

Full-length blots/gels are presented in the Supplementary file. The p-value was calculated using the two-sided Student's t-test: p>0.05 (ns), *p<0.05, **p<0.01, ***p<0.001, ****p<0.0001, vs control group. Immunohistochemistry and molecular analyses were performed using lung tissues from n=3–5 mice per group. Representative images and blots are shown. INOS: inducible nitric oxide synthase. TNF-α: tumor necrosis factor α. IL-1β: interleukin 1β. NLRP3: nucleotide-binding domain-like receptor protein-3. IL-6: interleukin 6. ARG-1: arginase-1. IL-10: Interleukin 10. NF-κB: nuclear factor kappa-B. p65: the p65 (RelA) subunit of NF-κB. p-p65: phosphorylation of p65. CS: cigarette smoke. ns: no significance.

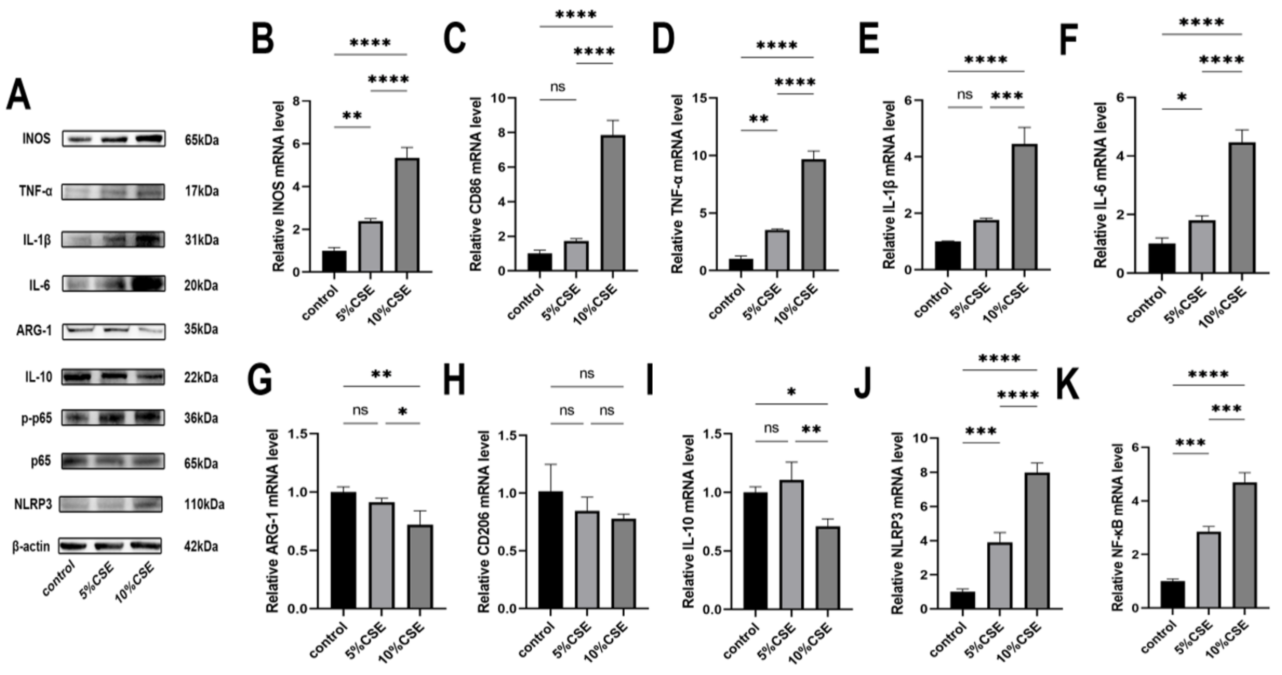

Supplementary Figure S2. Effect and mechanism analysis of CSE on inflammation and macrophage polarization in RAW 264.7 cells. Western blots analysis of INOS, TNF-α, IL-1β, IL-6, ARG-1, IL-10, p-p65, p65 and NLRP3 in RAW 264.7 cells (A). The mRNA expression of iNOS (B), CD86 (C), TNF-α (D), IL-1β (E), IL-6 (F), ARG-1 (G), CD206 (H), IL-10 (I), NLRP3 (J) and NF-κB (K) in mice

The p-value was calculated using the two-sided Student's t-test: p>0.05 (ns), *p<0.05, **p<0.01, ***p<0.001, ****p<0.0001, vs control group. Data represent three independent experiments using RAW264.7 cells. TNF-α: tumor necrosis factorα. IL-6: interleukin 6. IL-1β: interleukin 1β. ARG-1: arginase-1. NLRP3: nucleotide-binding domain-like receptor protein-3. INOS: inducible nitric oxide synthase. IL-10: Interleukin 10. NF-κB: nuclear factor kappa-B. p65: the p65 (RelA) subunit of NF-κB. p-p65: phosphorylation of p65. CSE: cigarette smoke extract. ns: no significance.

ARG-1	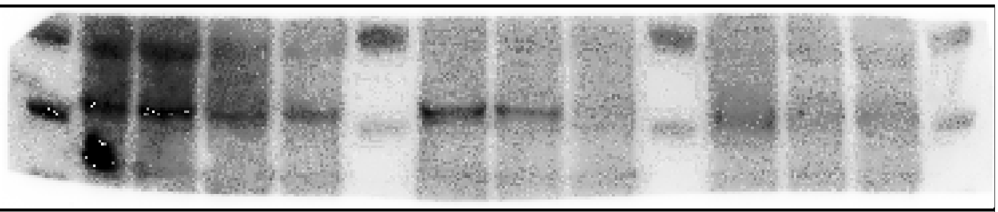
IL-1b	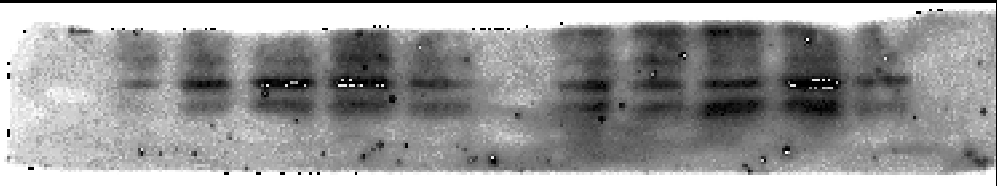
IL-6	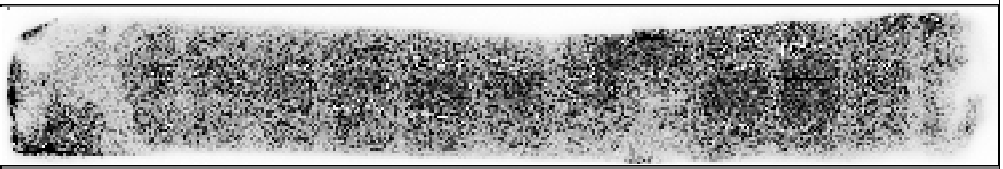
IL-10	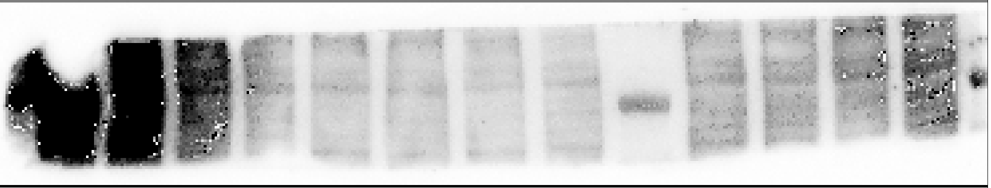
iNOS	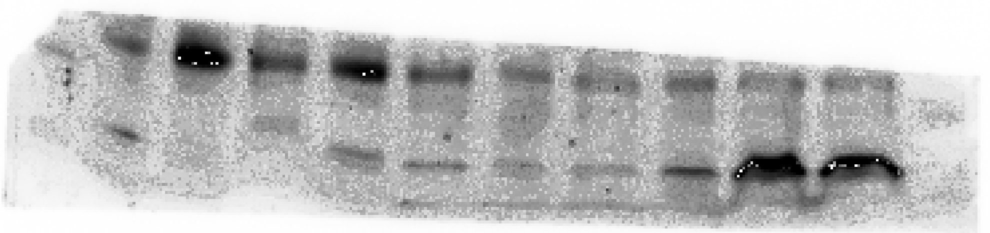
NLRP3	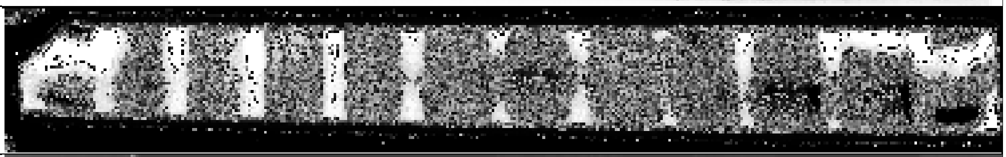
p65	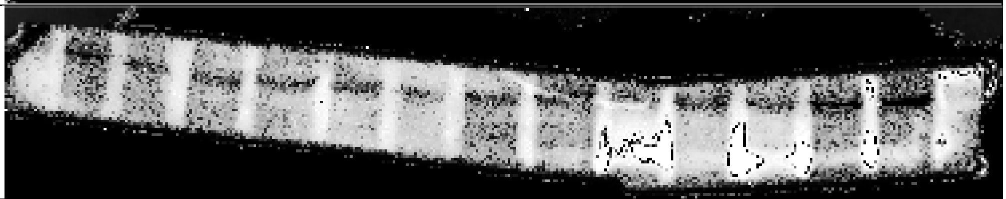
p-p65	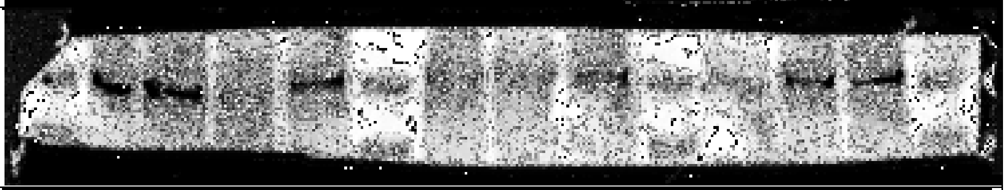
TNF-α	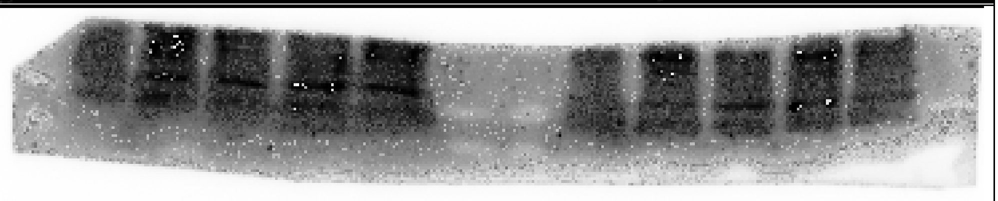

Actin	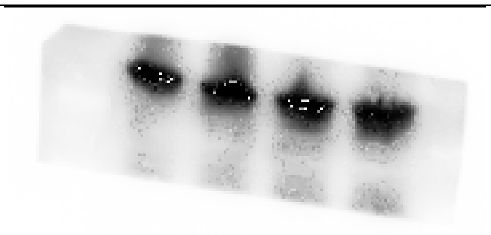

Supplementary Figure S3. Original Western blots

Western blots analysis of INOS, TNF-α, IL-1β, IL-6, ARG-1, IL-10, p-p65, p65 and NLRP3 in mice to study the effect and mechanism analysis of lung tissue inflammation and macrophage polarization in air-exposed mice (control) and CS-exposed (CS) mice.

ARG-1-RAW3	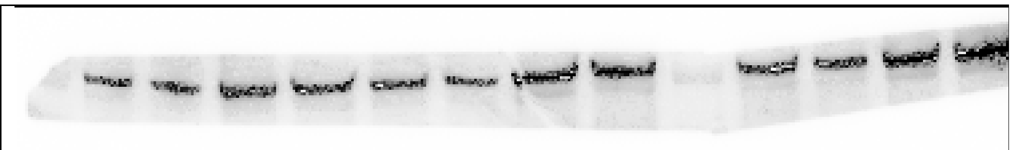
IL-1b-RAW3	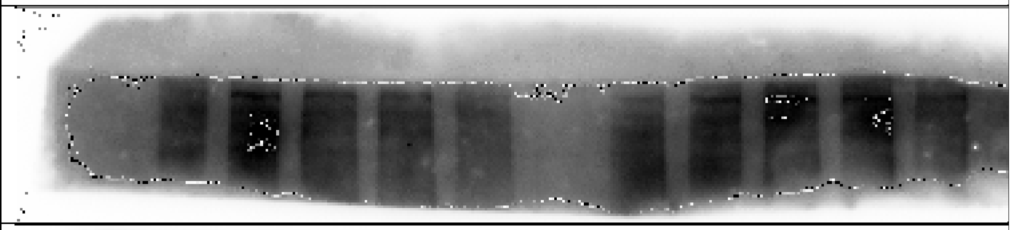
IL-6-RAW3	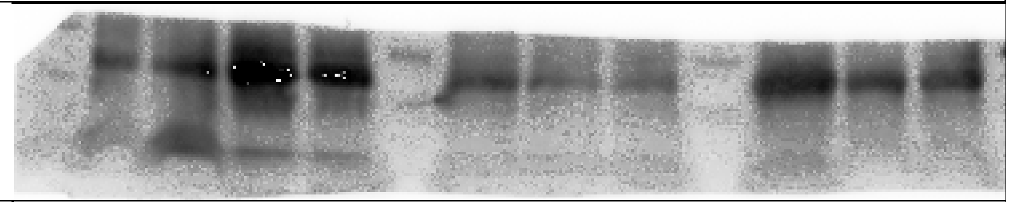
IL-10-RAW3	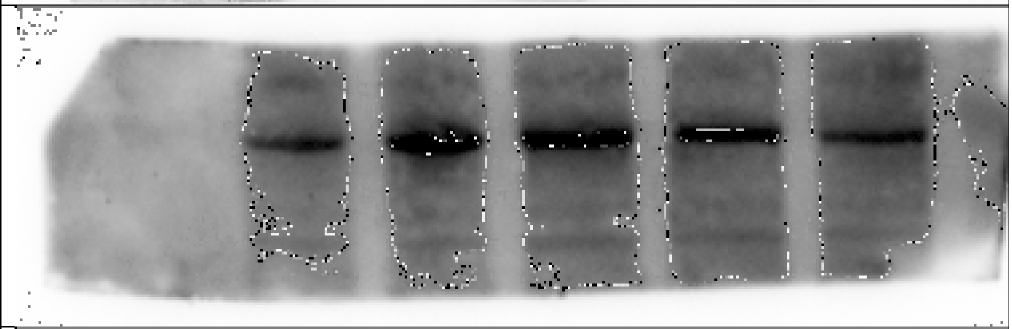
iNOS-RAW3	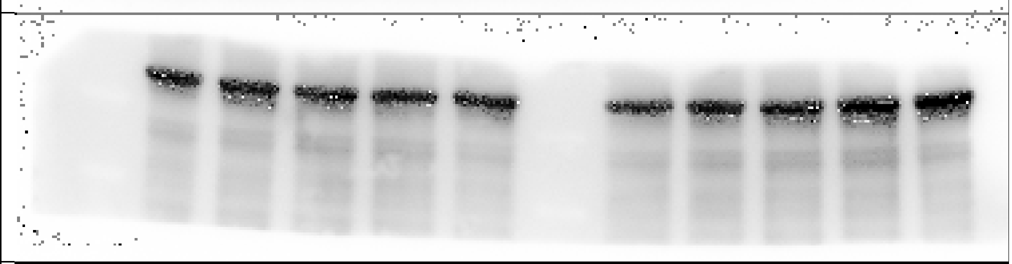
NFKB-RAW3	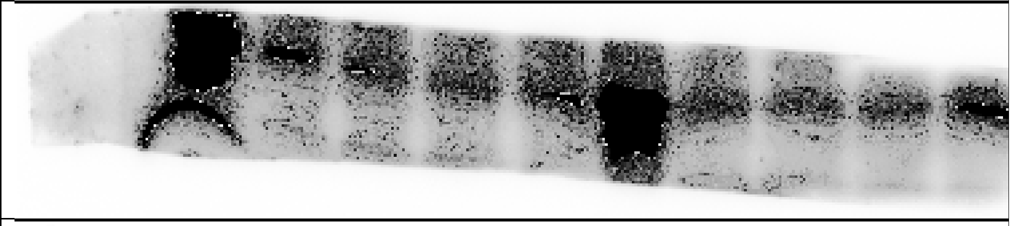
NLRP3-RAW3	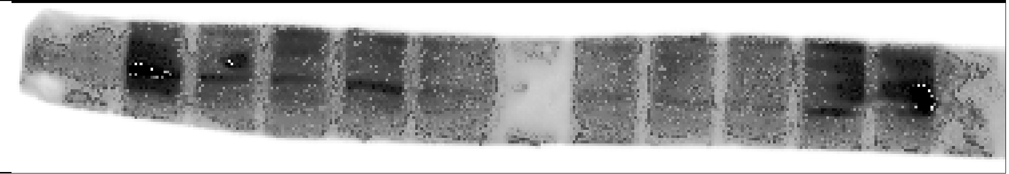

p-NFKB RAW3	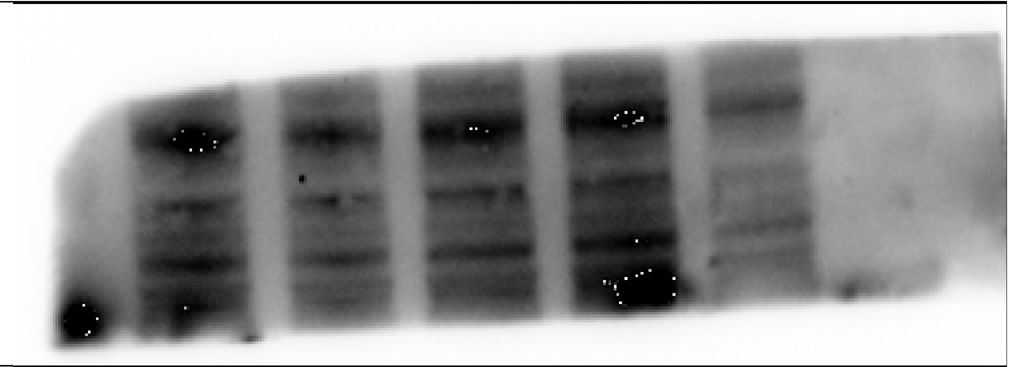
TNF-α-RAW3	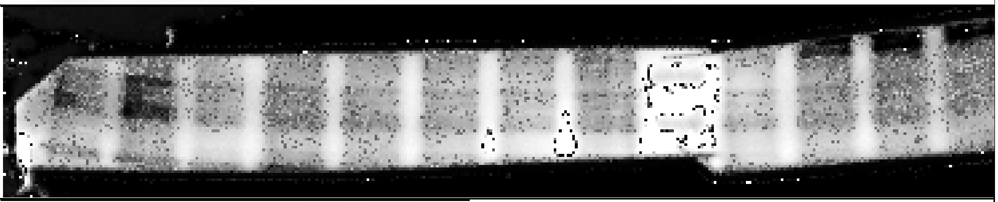
B-Actin-RAW3	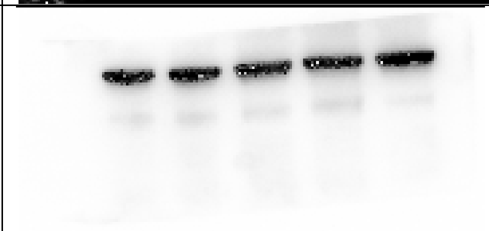

Supplementary Figure S4. Original Western blots

Full-length, uncropped Western blot images corresponding to the main figures are shown. Western blots analysis of INOS, TNF-α, IL-1β, IL-6, ARG-1, IL-10, p-p65, p65 and NLRP3 in RAW 264.7 cells to explore the effect and mechanism analysis of CSE on inflammation and macrophage polarization in RAW 264.7 cells. β-actin was used as the loading control and, where applicable, was derived from the same membrane used to detect multiple target proteins.

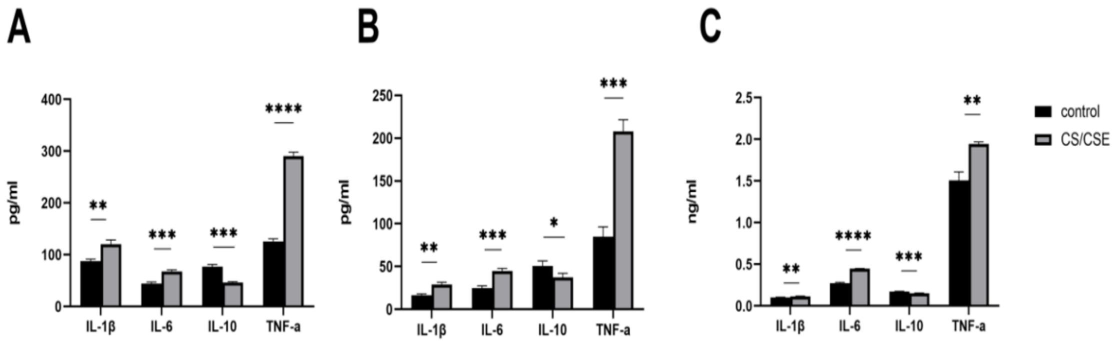

Supplementary Figure S5. The contents of TNF-α, IL-6, IL-1β, TGF-β and IL-10 detected by ELISA in BALF (A) and serum (B) of air-exposed mice (control) and CS-exposed (CS) mice, as well as in RAW 264.7 cells (C)

The p-value was calculated using the two-sided Student's t-test, ****$P < 0.0001$, vs. control group. For in vivo experiments, n = 3-5 mice per group. For in vitro experiments, data represent three independent experiments, each performed in triplicate.

Abbreviations: TNF-α: tumor necrosis factor α; IL-6: interleukin 6; IL-1β: interleukin 1β; TGF-β: transforming growth factor β; IL-10: Interleukin 10; CS: cigarette smoke; CSE: cigarette smoke extract.

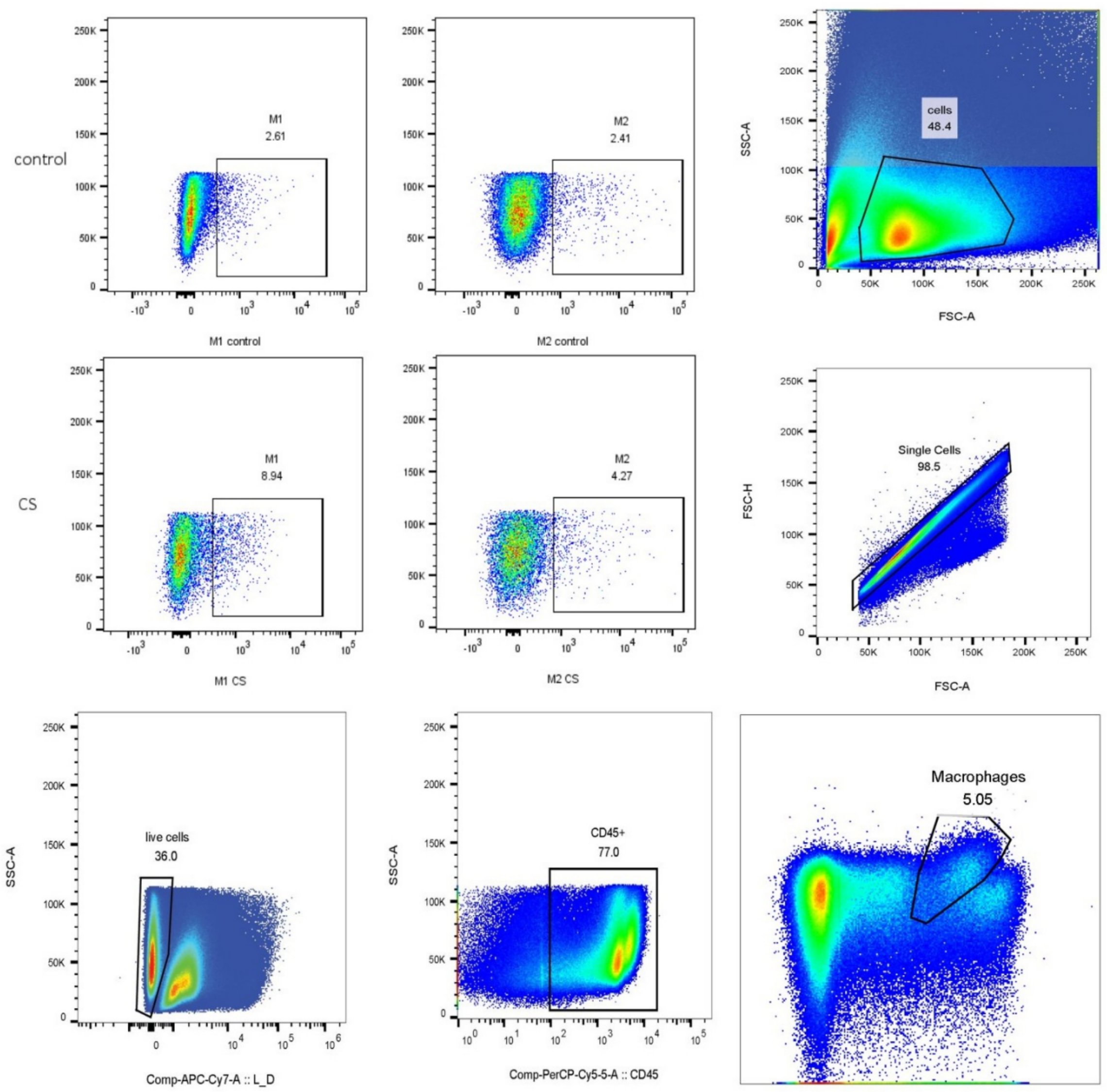

Supplementary Figure S6. Flow cytometric analysis of macrophage surface marker expression in lung tissues of mice and RAW264.7 cells

Flow cytometry gating strategy used to identify macrophages based on F4/80 expression and subsequent analysis of CD86 and CD206 surface marker expression in lung tissues. Statistical significance was determined using a two-sided Student's *t*-test ($P > 0.05$, ns; $P < 0.05$; $*P < 0.01$; $**P < 0.001$; $***P < 0.0001$ vs. control). For lung tissue flow cytometry, n = 3-5 mice per group. For RAW264.7 cell analyses, data represent three independent experiments.

Abbreviations: CS: cigarette smoke; CSE: cigarette smoke extract; ns: no significance.

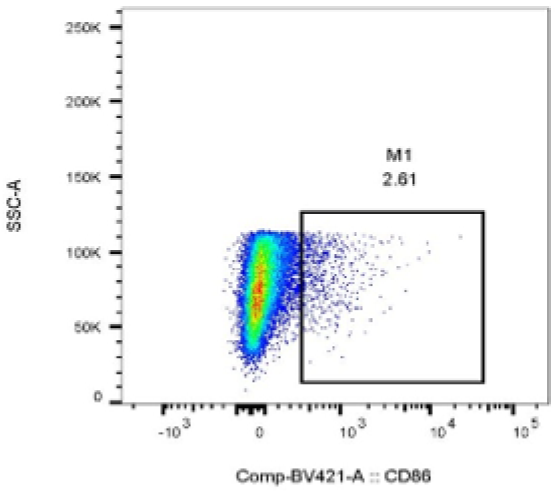
M1
2.61
SSC-A
Comp-BV421-A :: CD86
M2
2.41
Comp-PE-A :: CD206

A

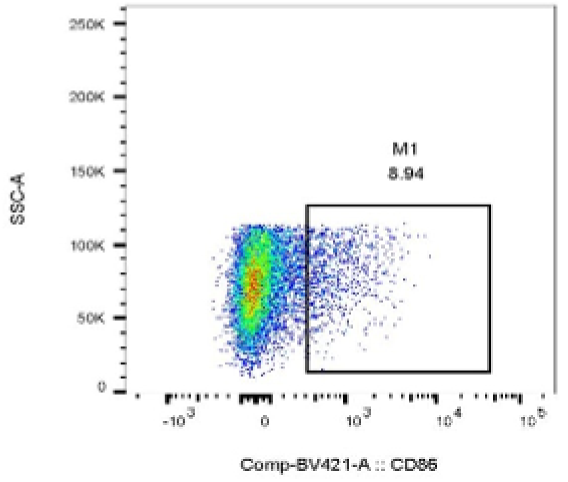
M1
8.94
SSC-A
Comp-BV421-A :: CD86
M2
4.27
Comp-PE-A :: CD206

A

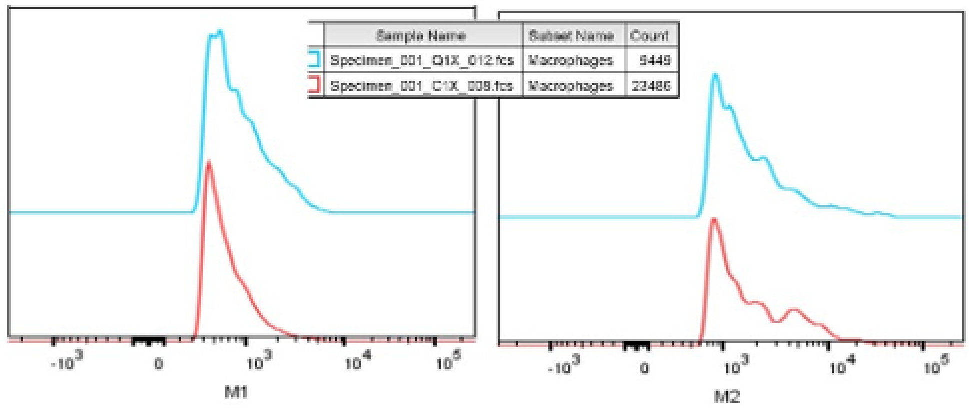
Sample Name | Subset Name | Count
Specimen_001_Q1X_012.fcs | Macrophages | 9449
Specimen_001_C1X_008.fcs | Macrophages | 23486
M1
M2

B

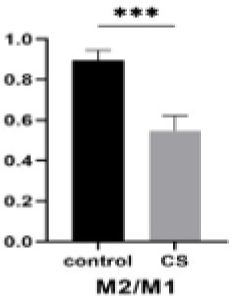
***
control
CS
M2/M1

C

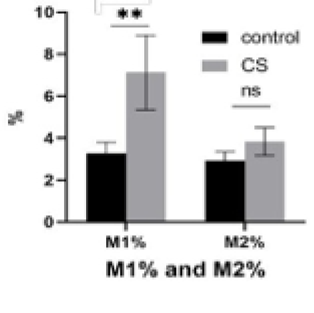
**
ns
control
CS
%
M1%
M2%
M1% and M2%

D

Supplementary Figure S7. Flow cytometric analysis of macrophage surface marker expression in lung tissues of mice and RAW264.7 cells

Representative FACS plots showing $CD86^+$ ($F4/80^+CD86^+$) and $CD206^+$ ($F4/80^+CD206^+$) macrophage populations in lung tissues of control and cigarette smoke (CS)-exposed mice (A, B). Quantitative analysis of the relative distribution of $CD206^+$ and $CD86^+$ macrophages, expressed as the ratio of $CD206^+/CD86^+$ cells (C), and the percentage of $CD86^+$ and $CD206^+$ macrophages within the total macrophage population (D) in mouse lung tissues. Statistical significance was determined using a two-sided Student's *t*-test ($P > 0.05$, ns; $P < 0.05$; $*P < 0.01$; $**P < 0.001$; $***P < 0.0001$ vs. control). For lung tissue flow cytometry, n = 3-5 mice per group. For RAW264.7 cell analyses, data represent three independent experiments.

Abbreviations: CS: cigarette smoke; CSE: cigarette smoke extract; ns: no significance.

Supplementary Table S1. List of primer for qRT-PCR.

Mouse Gene	Forward (5'-3')	Reverse (5'-3')
Nos2	GGAGTGACGGCAAACATGACT	TCGATGCACAACTGGGTGAAC
Cd86	CTGGACTCTACGACTTCACAATG	AGTTGGCGATCACTGACAGTT
Tnf	CCCTCACACTCAGATCATCTTCT	GCTACGACGTGGGCTACAG
Il1b	TCCAGGATGAGGACATGAGCAC	GAACGTCACACACCAGCAGGTTA
Il6	CCACTTCACAAGTCGGAGGCTTA	GCAAGTGCATCATCGTTGTTCATA C
Il10	CCCTTTGCTATGGTGTCCTT	TGGTTTCTCTTCCCAAGACC
Arg1	AGACAGCAGAGGAGGTGAAGAG	CGAAGCAAGCCAAGGTTAAAGC
Mrc1	CTGCAGATGGGTGGGTTATT	GGCATTGATGCTGCTGTTATG
Nlrp3	ATCAACAGGCGAGACCTCTG	GTCCTCCTGGCATACCATAGA
Gapdh	CGAAGCAAGCCAAGGTTAAAGC	CAGTGAGCTTCCCGTTCAGC
Nfkb1	AGAGGGGATTTCGATTCCGC	CCTGTGGGTAGGATTTCTTGTTC

The ARRIVE Guidelines Checklist

Animal Research: Reporting In Vivo Experiments

Carol Kilkenny[1], William J Browne[2], Innes C Cuthill[3], Michael Emerson[4] and Douglas G Altman[5]

[1]The National Centre for the Replacement, Refinement and Reduction of Animals in Research, London, UK, [2]School of Veterinary Science, University of Bristol, Bristol, UK, [3]School of Biological Sciences, University of Bristol, Bristol, UK, [4]National Heart and Lung Institute, Imperial College London, UK, [5]Centre for Statistics in Medicine, University of Oxford, Oxford, UK.

	ITEM	RECOMMENDATION	Section/ Paragraph
Title	1	Provide as accurate and concise a description of the content of the article as possible.	
Abstract	2	Provide an accurate summary of the background, research objectives, including details of the species or strain of animal used, key methods, principal findings and conclusions of the study.	
INTRODUCTION			
Background	3	a. Include sufficient scientific background (including relevant references to previous work) to understand the motivation and context for the study, and explain the experimental approach and rationale.<br>b. Explain how and why the animal species and model being used can address the scientific objectives and, where appropriate, the study's relevance to human biology.	
Objectives	4	Clearly describe the primary and any secondary objectives of the study, or specific hypotheses being tested.	
METHODS			
Ethical statement	5	Indicate the nature of the ethical review permissions, relevant licences (e.g. Animal [Scientific Procedures] Act 1986), and national or institutional guidelines for the care and use of animals, that cover the research.	
Study design	6	For each experiment, give brief details of the study design including:<br>a. The number of experimental and control groups.<br>b. Any steps taken to minimise the effects of subjective bias when allocating animals to treatment (e.g. randomisation procedure) and when assessing results (e.g. if done, describe who was blinded and when).<br>c. The experimental unit (e.g. a single animal, group or cage of animals).<br>A time-line diagram or flow chart can be useful to illustrate how complex study designs were carried out.	
Experimental procedures	7	For each experiment and each experimental group, including controls, provide precise details of all procedures carried out. For example:<br>a. How (e.g. drug formulation and dose, site and route of administration, anaesthesia and analgesia used [including monitoring], surgical procedure, method of euthanasia). Provide details of any specialist equipment used, including supplier(s).<br>b. When (e.g. time of day).<br>c. Where (e.g. home cage, laboratory, water maze).<br>d. Why (e.g. rationale for choice of specific anaesthetic, route of administration, drug dose used).	
Experimental animals	8	a. Provide details of the animals used, including species, strain, sex, developmental stage (e.g. mean or median age plus age range) and weight (e.g. mean or median weight plus weight range).<br>b. Provide further relevant information such as the source of animals, international strain nomenclature, genetic modification status (e.g. knock-out or transgenic), genotype, health/immune status, drug or test naïve, previous procedures, etc.	

Housing and husbandry	9	Provide details of:<br>a. Housing (type of facility e.g. specific pathogen free [SPF]; type of cage or housing; bedding material; number of cage companions; tank shape and material etc. for fish).<br>b. Husbandry conditions (e.g. breeding programme, light/dark cycle, temperature, quality of water etc for fish, type of food, access to food and water, environmental enrichment).<br>c. Welfare-related assessments and interventions that were carried out prior to, during, or after the experiment.	
Sample size	10	a. Specify the total number of animals used in each experiment, and the number of animals in each experimental group.<br>b. Explain how the number of animals was arrived at. Provide details of any sample size calculation used.<br>c. Indicate the number of independent replications of each experiment, if relevant.	
Allocating animals to experimental groups	11	a. Give full details of how animals were allocated to experimental groups, including randomisation or matching if done.<br>b. Describe the order in which the animals in the different experimental groups were treated and assessed.	
Experimental outcomes	12	Clearly define the primary and secondary experimental outcomes assessed (e.g. cell death, molecular markers, behavioural changes).	
Statistical methods	13	a. Provide details of the statistical methods used for each analysis.<br>b. Specify the unit of analysis for each dataset (e.g. single animal, group of animals, single neuron).<br>c. Describe any methods used to assess whether the data met the assumptions of the statistical approach.	
**RESULTS**			
Baseline data	14	For each experimental group, report relevant characteristics and health status of animals (e.g. weight, microbiological status, and drug or test naïve) prior to treatment or testing. (This information can often be tabulated).	
Numbers analysed	15	a. Report the number of animals in each group included in each analysis. Report absolute numbers (e.g. 10/20, not 50%[2]).<br>b. If any animals or data were not included in the analysis, explain why.	
Outcomes and estimation	16	Report the results for each analysis carried out, with a measure of precision (e.g. standard error or confidence interval).	
Adverse events	17	a. Give details of all important adverse events in each experimental group.<br>b. Describe any modifications to the experimental protocols made to reduce adverse events.	
**DISCUSSION**			
Interpretation/ scientific implications	18	a. Interpret the results, taking into account the study objectives and hypotheses, current theory and other relevant studies in the literature.<br>b. Comment on the study limitations including any potential sources of bias, any limitations of the animal model, and the imprecision associated with the results[2].<br>c. Describe any implications of your experimental methods or findings for the replacement, refinement or reduction (the 3Rs) of the use of animals in research.	
Generalisability/ translation	19	Comment on whether, and how, the findings of this study are likely to translate to other species or systems, including any relevance to human biology.	
Funding	20	List all funding sources (including grant number) and the role of the funder(s) in the study.	

References:
1. Kilkenny C, Browne WJ, Cuthill IC, Emerson M, Altman DG (2010) Improving Bioscience Research Reporting: The ARRIVE Guidelines for Reporting Animal Research. *PLoS Biol* 8(6): e1000412. doi:10.1371/journal.pbio.1000412
2. Schulz KF, Altman DG, Moher D, the CONSORT Group (2010) CONSORT 2010 Statement: updated guidelines for reporting parallel group randomised trials. *BMJ* 340:c332.
